# Responding to *Health Outcomes and Access to Health and Hospital Services in Rural, Regional and Remote New South Wales*

**DOI:** 10.1007/s11673-023-10237-8

**Published:** 2023-03-02

**Authors:** Fiona McDonald, Christina Malatzky

**Affiliations:** 1 Australian Centre for Health Law Research, Queensland University of Technology, Gardens Point Campus, 2 George Street, Brisbane, Queensland 4001 Australia; 2 Centre for Justice and School of Public Health and Social Work, Queensland University of Technology, Kelvin Grove Campus, 2 George Street, Brisbane, Queensland 4001 Australia

**Keywords:** Rural bioethics, Rural health ethics, Access to health services, Governance of health services, Australia, Rural health, Spatial inequities, Social justice, Structural violence

## Abstract

Ethical perspectives on regional, rural, and remote healthcare often, understandably and importantly, focus on inequities in access to services. In this commentary, we take the opportunity to examine the implications of normalizing metrocentric views, values, knowledge, and orientations, evidenced by the recent (2022) New South Wales inquiry into health outcomes and access to hospital and health services in regional, rural and remote New South Wales, for contemporary rural governance and justice debates. To do this, we draw on the feminist inspired approach to rural health ethics involving analysis of power relationships developed by Simpson and McDonald and related ideas from critical health sociology. In presenting this analysis, we extend contemporary thought about spatial health inequities and structural violence.

## Introduction

Events in New South Wales, Australia, have placed a spotlight on the (in)adequacies of health service provision to people living in regional, rural, and remote places in that state (Legislative Council New South Wales Portfolio Committee No. 2 – Health 2022) (NSW Report). The Committee’s conclusion that residents of regional, rural, and remote places in New South Wales have both poorer health outcomes and “inferior access to health and hospital services” compared to their metropolitan counterparts (NSW Report 2022, ix) was not a surprise. These findings are consistent with research and inquiry into the provision of health services to regional, rural, and remote regions in states, provinces, and nations around the globe (Scheil-Adlung 2015; Australian Institute of Health and Welfare, 2022; World Health Organization 2021). Ethical perspectives on these issues have, understandably and importantly, focused on inequities in access to services. In this commentary, we take the opportunity to examine the implications of normalizing metrocentric views, values, knowledge, and orientations (Roberts and Green 2013), evidenced in the NSW Report (2022), for contemporary rural governance and justice debates. To do this, we draw on the feminist inspired approach to rural health ethics involving analysis of power relationships developed by Simpson and McDonald (2017) and related ideas from critical health sociology. There are other lenses through which this type of analysis could be constructed, beyond “geographical narcissism” (Fors 2018), to reflect on structural violence against non-dominant groups. However, for the purposes of this paper we use a spatial analysis. In presenting this analysis, we extend contemporary thought about spatial health inequities.

## Metro-Normativity in the Health System—A Case for Rural Health Ethics Critique

Underlying all public policy are values and assumptions that require assessment (Sharpe 2004). Thus, ethics plays an important role in the justification and critique of public policy. From a sociological perspective, public policies are products of social institutions that, to be legitimized, must be perceived as acceptable and fair (Johnson, Dowd, and Ridgeway 2006; Woo, Ramesh, and Howlett 2017). In presenting healthcare systems as social institutions, Gilson (2003) argues that health systems—and, by extension, health policies—contribute to the (re)production of wider societal values, norms, and established social orders. We suggest that a key example of both this process and the need for rural health ethics critique is how values, experiences, and knowledge, grounded and produced in urban place contexts, are taken as the norm by health policy decision-makers, which perpetuates “unconscious beliefs that … power emanate[s] from the urban world” (Fors 2018, 446). For example, Ayres (1994) highlighted how clinical practice guidelines, regulated standards and accreditation standards are frequently formulated by metropolitan based health experts, who may not understand the practice style or resource constraints experienced within rural healthcare delivery (Simpson and McDonald 2017). This construction of power erases broader social consciousness about—and weakens sustained political will to address—the kinds of fundamental spatial health inequities exemplified in the NSW (2022) Report and others like it (Scheil-Adlung 2015; Rural Health Services Review Committee 2015; World Health Organization 2021). These fundamental spatial inequities are of chief concern. However, we acknowledge that spatial inequities in health outcomes between and within metropolitan and non-metropolitan areas are heterogeneous. This heterogeneity may, at least to a certain extent, reflect other differentials in, for example: level of need; socio-economic advantage; and risky behaviours (Australian Institute of Health and Welfare, 2022) as well as access related issues.

## Implications for Governance and Justice

The NSW Report (2022) demonstrates how metrocentric norms are built into governance structures, including those that are, to a certain extent, decentralized, in ways that undermine the ability of those in rural places to inform how healthcare is provided in-place. For example, local health districts in New South Wales are notionally designed to enable the needs of residents to be assessed by local actors. However, the operations and priorities of these districts remain implicitly regulated by the State and Federal governments through contracts, policy, and other directives. These levels of governance, located in the “ivory towers” (Simpson and McDonald 2017) of metropolitan Australia, undermine and inhibit the development of truly local governance and reinforce what Pesut, Bottorff, and Robinson (2011, 6) describe as a perception of “decision-making by strangers, at a distance.”

The literature on health system reform discusses the benefits and deficits of centralized versus decentralized models of health system governance (Sreeramareddy and Sathyanarayana 2019). However, in countries like Australia, this is usually at the level of central versus regional. In the main, more devolved mechanisms for health system governance at the local level are ignored (Simpson and McDonald 2017). When these are discussed, for example, with respect to Indigenous managed health services (metro or non-metro), ongoing practices of colonialization often silence or undermine comprehensive accounts centring on Indigenous perspectives—on what local governance can and should look like. This has implications, noted in part in the NSW Report (2022), for the provision of culturally (un)safe care and (dis)empowerment of local decision-makers. The NSW Report (2022) highlighted the impact of the hub and spoke model on regional, rural, and remote services. It was found that hubs (usually a regional centre) can be far removed from the concerns of people in smaller rural and remote places (those living nearer to the spokes) in their districts (s7.4), which enables the broader system and policymakers to effectively ignore these concerns.

The NSW Report (2022) discusses governance in terms of consultation with local communities and expresses uncertainty about whether this is done well (s. 7.4). However, in its broadest sense, justice includes participatory democracy, the belief that citizens and communities should be given opportunities to participate, more broadly than through consultation, in the governance of systems and structures that are important to them (Simpson and McDonald 2017). In the health context, the enterprise of a universal public health system builds on the value of solidarity. Citizens within a nation–state pool and re-distribute taxpayer funds to support each other to access the resources needed for health, including access to health services, and achieve broadly congruent health outcomes (Prainsack and Buyx 2015). This sense of solidarity and trust in the system can be undermined if a segment of the population does not benefit in an equitable way compared to others (Simpson and McDonald 2017).

One remedy is citizen engagement, in both metro and non-metro contexts. However, this may be even more relevant in regional, rural, and remote contexts where health services may be seen, by some, as central to the continuance and maintenance of their community (Simpson and McDonald 2017; Barnett and Barnett 2003). Also citizen engagement may serve a functional end: to challenge metrocentric norms in health service design and delivery and develop systems that are more adaptive to local conditions and better meet local needs. It also may serve a democratic end—to maximize citizen engagement in governance, which also supports the enactment of values such as solidarity. Further, citizen engagement addresses the aspect of realizing justice that relates to accountability. The capacity for both prospective and retrospective accountability is enhanced by broadening the role of public participation in local health service governance (Sharpe 2004). As Sharpe (2004, 14) notes, “prospective responsibility is oriented to the deliberative and practical processes involved in setting and meeting goals.” Thus, rural residents and communities need access to their own data to scrutinize the operations of local health services and discuss and contest the values underpinning service design as well as the pragmatics of opportunities/constraints in-place. Some mechanisms to increase citizen engagement suggested in the NSW Report (2022) include greater use of local engagement committees (Recommendation 42) and place-based needs assessments undertaken with input from communities (Recommendation 43). Other possibilities could include greater devolution of responsibilities to communities but only when those communities are willing and have the capacity to undertake this responsibility (Barnett and Barnett 2003).

Several obstacles are associated with realizing such a vision and are evident in the NSW Report (2022). One is the tension between the potential for local health systems governance and the purported need to ensure the maintenance of quality standards across the system (urban and regional/rural/remote) as a whole. Inherently, however, standards are imbued with societal norms, often urban-centric, which stifle place-conscious innovation (Simpson and McDonald 2017). The privileging of metrocentric knowledge and viewpoints within health systems and policy means that innovations driven from rural places, by rural knowledge and experiences, for rural places are difficult to progress and realize (Roberts and Green 2013). Given the recruitment and retention issues discussed extensively in the NSW Report (2022) and the literature more generally (Cosgrave 2020; World Health Organization 2021), such innovations are critical to improving access to rural health services and the health outcomes of people living outside of metropolitan places.

Concurrently, the true devolution of local governance in a neoliberal state is restricted by the choices of governments to under-resource local levels with the expectation that individuals and communities will fill the gaps left by state and federal government disengagement (Simpson and McDonald 2017). For example, the NSW Report (2022) raises concerns about the reliance on charity and community organizations to provide support and services (2.77). In the rural context, this sense that the community will be willing and able to fill the gaps may draw strength from the common stereotyping of the “rural idyll” and the construction of rural communities as self-reliant, self-sufficient, stoic, and resourceful (Malatzky and Bourke 2016; Simpson and McDonald 2017). This stereotype both shapes and justifies some of the assumptions underlying governance decisions affecting regional, rural, and remote health service design and delivery.

State resistance to local governance of health services by regional, rural, and remote communities may also stem from—and be justified by—stereotypes, common in highly urbanized Australia, that rural residents are inferior to those in metropolitan areas (Malatzky and Bourke 2016; Malatzky and Bourke 2018; Simpson and McDonald 2017). Here, urbanized spaces are constructed as “… progressive, where things happen and where diversity, excitement and innovation operate” (Malatzky and Bourke 2016, 161). Rural places, on the other hand, are often characterized as static or backwards looking. Further, rural health practice is popularly constructed as where health professionals who fail at urban practice go (Malatzky and Bourke 2016; Simpson and McDonald 2017). These dominant negative discourses centred on rurality and rural health support work are used to justify the metrocentric assumption that there is a lack of capacity for good governance within rural communities. This is despite examples of flourishing rural health (Barnett and Barnett 2003) and social enterprises and businesses in some, but not all, rural places, indicating motivation and capacity. Simpson and McDonald (2017) argue this stereotyping has substantial implications for justice and, as such, there is a moral imperative to deconstruct its impact on the practices of health system governance.

## Extending the Conceptual Tools for Rural Health Ethics Critique

In critiquing how neoliberal logics inform decision-making processes in health, Paul Farmer argued that dismantling the deep causes of health inequities—the fundamental barriers to justice—should be the central objective of healthcare. Farmer and colleagues’ (2006) concept of structural violence offers a potentially powerful interpretation of what the health inequalities and human indignities experienced within rural health services and communities detailed in the NSW Report (2022) represent. Structural violence is understood as “social arrangements that put individuals and populations in harm’s way … The arrangements are structural because they are embedded in our social world’s political and economic organization; they are violent because they cause injury to people” (Farmer et al. 2006, 1686). The embeddedness of metro-normativity within health and broader social systems, including political and economic structures and social imaginaries, position rural people at a considerable disadvantage in contests over political control of resources and place autonomy (Malatzky et al. 2020). The effects on access to health services and health outcomes, exemplified in the NSW Report (2022) cannot be considered as anything other than injurious for rural people and communities. According to De Maio (2015, 680), structural violence is a “multi-level idea, through which different ‘axes’ of oppression … may intersect to generate preventable morbidity and premature mortality in marginalised populations.” Following Bambra’s (2022) argument for place to be conceptualized as an aspect of intersectionality, rurality is an element of social classification that can interlock with others in matrixes of discrimination (Cho et al. 2013) and contribute to the reproduction of health inequalities. Such inequalities result from social arrangements that cause suffering for groups of people, and for that suffering to go largely unnoticed by those whose interests are well served by current arrangements (Farmer 2003). Conceptualizing the systemic disadvantaging of rural residents within contemporary social systems as a form of structural violence could be productive for extending rural health ethics critiques focused on power and social justice. Within a framework of rural health ethics, such conceptualizations illuminate the structural mechanisms through which metro-normativity affects rural health and impedes the acquisition of justice for local communities. It may also contribute to the development of ethically conscious socio-structural and political solutions.

## Conclusion

The NSW Report (2022) illustrates what spatial inequities mean for regional, rural, and remote residents of NSW when trying to access, or accessing, health services. This report is an account of inequalities of access playing out across a swathe of the population in NSW. It also raises significant questions about the extent to which the metro-normativity embedded into governance structures constitutes a form of structural violence against residents of regional, rural, and remote regions. In this commentary, we argue that there may be metro-normative values, assumptions, and presumptions underlying governance practices supporting the delivery of health services to regional, remote, and rural populations in NSW and, more broadly, require assessment and critique. Equity is not only about outcomes; it is also about deliberative and transparent processes that recognize diversity and strengths that can emerge outside the metropolis.
